# Complete Genome Sequence of *Cellulophaga lytica* HI1 Using PacBio Single-Molecule Real-Time Sequencing

**DOI:** 10.1128/genomeA.01148-14

**Published:** 2014-11-06

**Authors:** Audrey Y. Asahina, Michael G. Hadfield

**Affiliations:** Pacific Biosciences Research Center Kewalo Marine Laboratory, University of Hawaii at Manoa, Honolulu, Hawaii

## Abstract

We report here the complete genome sequence of *Cellulophaga lytica* HI1 isolated from a seawater table located at the Kewalo Marine Laboratory (Honolulu, HI). This is the first complete *de novo* genome assembly of *C. lytica* HI1 using PacBio single-molecule real-time (SMRT) sequencing, which resulted in a single scaffold of 3.8 Mb.

## GENOME ANNOUNCEMENT

*Cellulophaga lytica* HI1 was originally isolated from a seawater table at the Kewalo Marine Laboratory (Honolulu, HI) (1) and identified through 16S rRNA sequencing. This is the third completed genome sequenced from a member of the genus *Cellulophaga*. The previous two completed genomes used hybrid sequencing and are *C. lytica* type strain LIM-21ta (2), isolated from a marine mudflat in Costa Rica, and *Cellulophaga algicola* (3).

*C. lytica* (phylum *Bacteroidetes*, family *Flavobacteriaceae*) is a yellow/orange, aerobic, agarolytic, Gram-negative rod, which displays gliding motility. Microorganisms in the family *Flavobacteriaceae* can be found in a wide range of habitats, which include terrestrial, fresh, and marine water environments. *C. lytica* is known to have enzymatic activity that can lead to the lysis of eukaryotic organisms, such as the toxic dinoflagellate *Gymnodinium catenatum* (4), and is a novel source of biosurfactants (5). Other enzymes that are produced by *C. lytica* are noted to degrade carrageenan, a compound found in many species of red seaweed (6). Additionally, *C. lytica* is also just one of a host of biofouling microorganisms that can be found in marine biofilms (1). As a primary microbial biofouler, *C. lytica* HI1 has been shown to be moderately effective at inducing the settlement and metamorphosis of the serpulid polychete *Hydroides elegans* (1), a major marine biofouling organism. Thus, as a biofouling microorganism, *C. lytica* is often used to assess the effectiveness of antifouling and foul release coatings (7, 8).

DNA was extracted using the MoBio UltraClean microbial DNA isolation kit submitted to the National Center for Genome Resources (NCGR) for PacBio single-molecule real-time (SMRT) sequencing. A single library was prepared for *C. lytica* HI1 and run on 2 SMRT cells. With a genome size of approximately 3.8 Mb, PacBio SMRT sequencing provided approximately 100× coverage of the entire *C. lytica* HI1 genome. SMRT sequencing of the *C. lytica* HI1 genome initially resulted in 156,902 raw reads, with a mean read length of 5,564 bp, totaling 873,038,511 nucleotides. The generated reads were then introduced into the Hierarchical Genome Assembly Process (HGAP), which includes assembly with the Celera Assembler and assembly polishing with Quiver. The final complete genome resulted in a single scaffold of 3,824,196 bp, with a total G+C content of 32%. The completed genome was annotated by the NCBI Prokaryotic Genome Annotation Pipeline and Rapid Annotations using Subsystems Technology (RAST) server (9, 10) and manually curated with GenePRIMP (11). RAST predicted 3,396 coding sequences, of which 49 encode RNA regions. Specifically, of the 49 RNA regions, 7 encode rRNA and 41 encode tRNA. Six phage components were also identified by RAST (i.e., phage tail fiber protein, prophage/phage protein, and phage integrase). A single confirmed and 5 putative clustered regularly interspaced short palindromic repeat (CRISPR) regions were also identified (http://crispr.u-psud.fr/). antiSMASH (12) analysis for the identification of secondary metabolites predicted one gene cluster encoding the metabolite terpene. The complete genome sequence of *C. lytica* HI1 will allow for the mining of genes coding for potentially useful natural products and secondary metabolite production that are of biological or biotechnological importance.

### Nucleotide sequence accession number.

The complete genome of *C. lytica* HI1 has been deposited in the NCBI database under the accession no. CP009239.
